# A case report of acute hypothermia during initial inpatient clozapine titration with review of current literature on clozapine-induced temperature dysregulations

**DOI:** 10.1186/s12888-020-02695-w

**Published:** 2020-06-09

**Authors:** Bradley G. Burk, Alex H. Ward, Brooke Clark

**Affiliations:** 1 Department of Pharmacy, University of Alabama at Birmingham Medical Center, JT1728 619 19th Street South, Birmingham, AL 35249 USA; 2 Chattanooga College, 5600 Brainerd Road, Chattanooga, TN 37415 USA

**Keywords:** Clozapine, Hypothermia, Temperature, Schizophrenia, Schizoaffective, Antipsychotic

## Abstract

**Background:**

Here we describe a unique case of clozapine-associated hypothermia during initial titration of this medication in an acute inpatient psychiatry setting. Only a handful of cases on this topic have been published. We discuss possible pharmacologic mechanisms supporting or refuting the propensity of clozapine to induce hypothermia, as well as risk factors for clozapine-induced hypothermia, and a comparison to clozapine-induced hyperthermia.

**Case presentation:**

A 70 year-old African American female with treatment-refractory schizoaffective disorder developed hypothermia with a nadir temperature of 89 °F (31.7 °C) after 7 days on clozapine, on a total dose of 50 mg twice daily. Accompanying symptoms included bradycardia, hypotension, QTc prolongation, tachypnea, hypoxemia, and an absence of shivering. The patient was transferred to the ICU, and rewarmed within 10 h with the discontinuation of her clozapine, ziprasidone, and carvedilol. Broad spectrum antibiotics were initiated, but discontinued shortly after, as the patient had no leukocytosis, and blood cultures were negative.

**Discussion:**

While hypoglycemia, hypothyroidism, sepsis, and stroke were effectively ruled out, alternative drug-disease (including chronic kidney disease), and drug-drug interactions were considered possible contributing features. Benzodiazepines, valproic acid, ziprasidone, and the numerous antihypertensive agents the patient was taking were considered as either primary or compounding factors for hypothermia. After exclusion or inclusion of these alternative causes, we calculated a score of 4 (possible) for clozapine-induced hypothermia on the Naranjo Scale.

**Conclusions:**

Clozapine-induced hypothermia may occur more commonly than clinicians believe. Practitioners should be cognizant of this potentially fatal phenomenon, and monitor for temperature dysregulations while on clozapine, especially during initial titration, in those with multiple comorbid factors, and on additional medications that may contribute to hypothermia.

## Background

While it is well established that antipsychotics may induce hyperthermia (an often-recognized part of neuroleptic malignant syndrome), hypothermia from these agents may be vastly under-recognized and under-represented within medical literature [1]. It was with the advent of chlorpromazine in the 1950s that the first case report of phenothiazine-induced hypothermia was published [2]. In an older review of neuroleptic-induced temperature dysregulations using primarily first-generation agents in various species, the authors found these agents decreased body temperature in 321 of 651 studies, and increased it in 183 cases [3]. However, of the studies specifically in humans, temperature decreased in only 26 of 153 cases, and increased in 127 cases [3]. A lack of systematic reviews with newer antipsychotics has left a gap in knowledge.

A publication from 2017 examined original case reports of possible antipsychotic-induced hypothermia [4]. The risk of hypothermia appears to be highest during the first 7 days of therapy, but may occur after the first dose [1, 4, 5]. Additional factors, such as advanced age, cold exposure, use of benzodiazepines, and hypothyroidism (subclinical), were also noted to predispose a patient on antipsychotics to hypothermia [1, 6]. Of the 57 case reports analyzed, 52% were with second-generation antipsychotics [4]. These second-generation antipsychotic-induced hypothermia cases were further investigated in a more recent review, which found olanzapine and risperidone to be the most common causes of hypothermia [7]. Based on the comparatively larger number of hypothermia reports to drug-monitoring agencies such as Food and Drug Administration (FDA) Medwatch and World Health Organization (WHO), these authors also concluded that the incidence of hypothermia is at least 10 times higher than is indicated by published literature [4, 6, 7]. Indeed, if one were to compare the 591 cases of hypothermia with second-generation antipsychotics reported to the FDA to the 34 published case reports, this estimate seems highly conceivable [7].

Although one report speculated clozapine may be safely utilized in those with a history of hypothermia from alternative antipsychotics, newer reports suggests this may not be the case [8, 9]. Here we present a unique case of hypothermia occurring on initial titration of clozapine during an inpatient psychiatry admission. We also aim to provide clinicians with greater insight into clozapine-induced temperature dysregulations.

## Case presentation

Ms. P is a 70-year-old African-American female with a history of schizoaffective disorder (bipolar type), vascular dementia, hypertension, coronary artery disease, chronic heart failure, dissected ascending aortic aneurysm, and chronic kidney disease (CKD) admitted to inpatient psychiatry for symptoms of mania and delusions. Her hospital admission was complicated by profound hypertension, acute kidney injury, and an acute on chronic heart failure exacerbation.

Records indicated Ms. P was prescribed risperidone prior to her hospitalization, which was restarted upon admission at a dose of 1.5 mg daily, titrated to 4 mg daily and continued for 3 weeks. Her risperidone was subsequently switched to olanzapine 5 mg daily, which was titrated to dose of 20 mg daily and continued for 2 weeks, then later changed to ziprasidone 40 mg twice daily. Due to inadequate response to all of these agents, she was ultimately placed on clozapine for her refractory schizoaffective symptoms. At this time, she was kept on ziprasidone with anticipation of tapering off after titrating clozapine to an adequate dose. The clozapine was started at a dose of 12.5 mg twice daily, titrated up to 25 mg twice daily after six doses, then up to 50 mg twice daily after three doses. The patient’s medication list during hospitalization is located in Table 1.

**Table 1:** Prescribed medications and corresponding doses Ms. P was taking at the time of hypothermia onset

Aspirin 81 mg PO daily	Ferrous Sulfate 650 mg PO TID	Clozapine 50 mg PO BID	Multivitamin PO daily
Atorvastatin 40 mg PO daily	Clonidine 0.3 mg/24 h transdermal patch	Divalproex Sodium ER 1250 mg PO nightly	Donepezil 10 mg PO daily
Brimonidine 0.15% eye drops daily	Heparin 5000 units SQ TID	Docusate 200 mg PO daily	Trazodone 100 mg PO PRN HS
Amlodipine 10 mg PO daily	Hydralazine 100 mg PO TID	Ziprasidone 100 mg PO daily	Acetaminophen 650 mg PO PRN QID
Carvedilol 25 mg PO BID	Isosorbide dinitrate 60 mg PO BID	Lorazepam 1 mg PO TID	

**Fig. 1:**
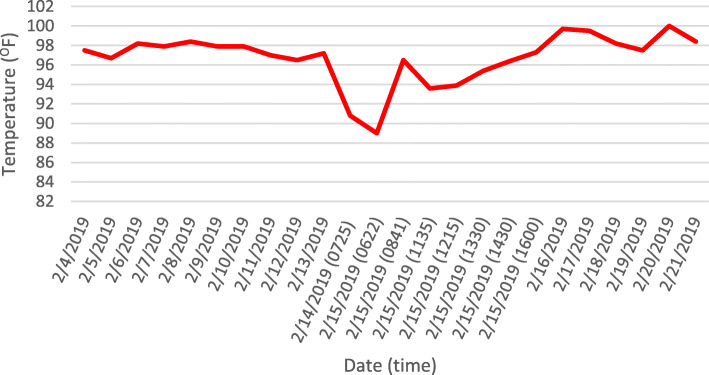
Temperature (°F) log prior to and during hypothermic event

On day 50 of admission, Ms. P developed hypothermia, with a temperature of 90.8 °F (32.7 °C). By the next morning (day 7 of clozapine trial), she displayed hypoxemia (O_2_ saturation 88%), tachypnea, and worsening hypothermia (Fig. 1). While the patient had been intermittently bradycardic prior to this, her heart rate was lower during this event, ranging between 40 and 50 beats per minute. The patient displayed both hypotension as well as hypertension during this event, with blood pressure readings between 96 and 153 mmHg systolic and 39–75 mmHg diastolic. She appeared somnolent and confused at this time, and remarked that she was cold (and had cold extremities to touch); however, no shivering was noted on exam.

Due to concerns for sepsis, Ms. P was transferred to the intensive care unit, where she became normothermic within approximately 10 h with the use of warming blankets, and the discontinuation of her clozapine, ziprasidone, and carvedilol. Laboratory values were remarkable for elevated procalcitonin (0.61 ng/mL), hypoxemia, and hypercapnia, while other values were within normal limits (Table 2). Broad-spectrum antibiotics were initiated, but were discontinued after 3 days as the patient had no leukocytosis, and blood cultures were negative. Her electrocardiogram was significant for bradycardia with vagally mediated pauses, and a prolonged QTc (505 milliseconds), but no Osborn J-waves were apparent. An echocardiogram conducted did not find any valvular vegetation, but noted a reduced ejection fraction of 49% (baseline 55%). A prior CT of the head within the previous 4 months displayed chronic lacunar infarcts within the left thalamus and right centrum semiovale, but no acute abnormalities or changes. A repeat CT scan was not performed during her hypothermic episode.

**Table 2:** Laboratory test results performed at time of hypothermia onset

Sodium 142 mMol/L	Serum Creatinine 1.5 mg/dL (H)	Bicarbonate 30 mMol/L	Magnesium 2.3 mg/dL	Potassium 4.2 mMol/L	Chloride 103 mMol/L	Amylase 50 units/L
Phosphate 3.7 mg/dL	Calcium 8.8 mg/dL	BUN 38 mg/dL (H)	RBC 2.83 × 10^6^/cmm (L)	Hemoglobin 8.3 g/dL (L)	Hematocrit 25% (L)	Urinalysis NEG
WBC 4.42 × 10^3^/cmm	MCV 89 fL	Platelets 94.2 × 10^3^/cmm (L)	Albumin 3.3 g/dL (L)	TSH 4.62 mInt-units/mL	Free T4 0.66 ng/dL	Blood cultures NEG
Glucose 70 mg/dL	Procalcitonin 0.61 ng/mL (H)	Troponin < 0.030 ng/mL	Alk Phos 96 units/L	AST 29 units/L	ALT 43 units/L	Viral Panel NEG

## Discussion

Other considerations of secondary hypothermia included hypothyroidism, hypoglycemia, stroke, and sepsis. However, as indicated in Table 2, the patient’s thyroid stimulating hormone (TSH) and blood glucose drawn at the time of the hypothermic episode were within normal limits. As the patient did not present with leukocytosis and all blood cultures and viral panels returned negative, sepsis was ruled out as a likely cause of the hypothermia. Although altered mental status was noted, stroke was excluded, as neurologic exam was grossly normal. In addition, while we recognize antihypertensives as a potential cause, secondary to their ability to inhibit vasoconstriction and shivering, we do not believe these were the sole reason behind her hypothermia. Rather, these agents might have compounded the effects brought on by clozapine. Our rationale may be supported by the fact that the patient had not received two of her carvedilol doses before the hypothermic episode secondary to bradycardia, and because Ms. P had been on the majority of these antihypertensives (save isosorbide dinitrate) for a prolonged period prior to this hypothermia episode.

Alternative drug-disease interactions must also be considered a risk factor for hypothermia in this patient. Clozapine (along with all other antipsychotics) possesses a black box warning for all-cause mortality in those with dementia. This risk appears to be consistent even for those specifically with vascular dementia [10]. It is evident based on the current data that those with dementia are more highly susceptible to the adverse effects (possibly including hypothermia) of antipsychotics. Acute heart failure exacerbations may also precipitate hypothermic events, secondary to inadequate oxygen delivery in the body, and may have been a causative factor in our patient [11]. However, the inverse of this might also be true, as hypothermia may induce or exacerbate heart failure [12]. The patient’s history of CKD should also be taken into account. While the manufacturer lists no specific dose adjustments for clozapine in those with renal dysfunction, only 0.55% is recovered within the urine unchanged, with 48.5% excreted as non-pharmacologically active metabolites [13]. Dose reductions of clozapine may be a necessity in those with renal dysfunction.

Ms. P was taking additional psychotropics aside from clozapine, all of which have case reports of hypothermia, or are thought to magnify the risk of hypothermia (Table 1). Although the literature appears varied, divalproex may significantly increase the levels of clozapine [14, 15]. While divalproex was the primary agent considered to have caused hypothermia in some of the available reports, it should be noted that data exists on hypothermic resolution after antipsychotic cessation in the presence of continued divalproex use [16]. As previously stated, our patient was kept on divalproex, with no further episodes of hypothermia during her stay, leading us to rule out divalproex as the sole cause of her hypothermia. Although cases of using ziprasidone safely in those with a history of hypothermia exist, other cases portray ziprasidone-induced hypothermia occurring even after 3 years on this medication [17]. Thus, we cannot fully rule out ziprasidone as a potential cause of hypothermia in our patient. Benzodiazepines, such as the lorazepam prescribed to our patient, are thought to inhibit the preoptic nucleus, preventing vasoconstriction and shivering [18]. Because nearly 80% of case reports denote hypothermia generally occurs after drug initiation or dose increases, we considered clozapine as the most likely cause in our patient [1, 7]. After exclusion or inclusion of these alternative causes, we calculated a score of 4 (possible) for clozapine-induced hypothermia on the Naranjo Scale for determining the probability of an adverse drug reaction (scoring “yes” on questions 1, 2, 3, 5, and 10) [19].

It should be acknowledged that Ms. P has never had pharmacogenomic testing performed. Owing to the fact that clozapine is highly metabolized by cytochrome P450 (CYP) 1A2 and CYP2D6, we cannot discount that a genetic polymorphism placed this patient at increased risk for hypothermia secondary to higher than intended levels. As depicted in Table 1, the patient was not on any medications known to inhibit CYP1A2 or CYP2D6. A clozapine level was not performed at the time of hypothermia to determine if the level was supratherapeutic. Also of interest, pharmacokinetic and pharmacodynamic processes are altered in hypothermia. CYP450 metabolizing enzymes (including CYP3A4 and CYP2D6) are noted to be less active during hypothermia, leading to decreased clearance of the medications they metabolize [20]. Though unclear, it is possible the effects of a medication leading to hypothermia may be amplified in such an environment, if that particular medication is metabolized through a CYP450 pathway.

## Literature review

Only a handful of case reports on clozapine-induced hypothermia exist, with varying onset and doses depicted. Papazisis et al. described a 71-year old male individual with hypothermia and accompanying Osborn J-waves after a year on 300 mg/day of clozapine [21]. This patient was switched to quetiapine with no further incidence of hypothermia [21]. Pelechas et al. described a 61-year old male taking clozapine, diazepam, and haloperidol who developed hypothermia after an unknown period [22]. A more recently published case series reported a 65-year old male who developed mild hypothermia after 5 days of clozapine use, on a total dose of 50 mg/day, which subsided after discontinuing clozapine and switching to olanzapine [4]. These authors also reported a 59-year old female with hypothermia lasting more than 6 days while on clozapine, risperidone, and olanzapine [4]. The authors appear to attribute this case primarily to risperidone, as this agent was recently titrated, and because hypothermia persisted even after discontinuing the olanzapine and clozapine [4]. In a case of intentional overdose on 3.5 g of clozapine (concentration 2183 ng/mL), the patient was noted to have a temperature of 91.6 °F (33.1 °C) upon admission [23].

We extracted data on clozapine and hypothermia from the FDA Adverse Event Reporting System (Table 3) [24]. Data from 1990 to 2019 demonstrated 109 separate cases of individuals experiencing hypothermia while taking clozapine. Clozapine was the primary suspect in 83 of these cases. The majority of cases were considered serious, and 21 patients died after exposure. Schizophrenia was by in large the most common indication for use of clozapine. Table 3 provides information on the onset of hypothermia after initiation of clozapine. While the single largest percentage of cases occurred within the first month (~ 27%), it is important to note that the majority of cases happened within 1 year of being on this medication, and even after 10 years on clozapine. However, it is unknown as to whether or not cases occurring later in therapy happened after any dose increases. Of those with clozapine-induced hypothermia, 60% were male. Middle to advanced age appeared more highly correlated with clozapine-induced hypothermia (63% for ages 40+, with 25.8% aged 60+), with the average age of 46 years. Haloperidol, antidepressants (any), valproic acid, olanzapine, and benzodiazepines (or any “z” drugs), were among the top co-prescribed medications for those with clozapine-induced hypothermia (9.2, 11, 11, 12, and 17.4% respectively). When co-administered with clozapine, event reporters considered valproic acid a suspect of hypothermia in 4 (15.4%) cases, benzodiazepines were considered suspects in 5 (19.2%) cases, while olanzapine was considered a suspect in 8 (30.7%) cases. Interestingly, the demographics and characteristics within Table 3 fit relatively closely with those depicted in a publication from Windfuhr et al., wherein the incidence of sudden unexplained death (SUD) from antipsychotics was examined [25]. In this paper, the authors found that older male patients were slightly more at risk of SUD (58% vs. 42% female), with other risk factors including clozapine, benzodiazepines, cardiovascular disease, and a diagnosis of dementia [25]. This correlation may point to hypothermia as a potential cause of SUD from antipsychotics.

**Table 3:** Demographics & Characteristic Features of Clozapine-Induced Hyper- and Hypothermia

Demographics & Characteristic Features	Clozapine-Induced Fever	Clozapine-Induced Hypothermia
**Onset**	< 1 month appears to be greatest risk, but may occur at any time or with dose increases [24–26]	
**Dose Related**	Unlikely, but risk possibly increased with overdose [23, 24]	
**Titration Related**	Possibly (dose increases > 50 mg/week) [27, 28]	Unknown
**Concomitant Medication(s) Which May Increase Risk**	Valproic acid, additional antipsychotics [28, 29]	Valproic acid, benzodiazepines, additional antipsychotics [24]
**Sex Category**	Male>female (~ 1.5:1) [24]	
**Age (years)**	Average age ~ 41 (~ 70% are 40+, with ~ 5.4% being 60+) [24, 27]	Average age ~ 46 (~ 63% are 40+, with ~ 25.8% being 60+) [24]
**Seen on Rechallenge**	Possibly, but uncommon [27, 28]	Unknown

In a 2007 review, van Marum et al. found 524 cases of hyperthermia during antipsychotic use reported to the WHO international database for Adverse Drug Reactions [1]. This was surprisingly similar to the number of hypothermia cases reported (n = 480). The incidence of NMS from antipsychotics in recent studies ranges from 0.01–0.02%, while earlier studies reported incidences between 0.2–3.23% [29, 30]. This decline in incidence may represent an increased awareness by practitioners. NMS caused by antipsychotics has been postulated to be secondary to potent D2 antagonism, and thus considered more common with the first-generation antipsychotics; although this remains uncertain, as virtually every antipsychotic has had case reports depicting NMS [30]. Even in the absence of frank NMS, some antipsychotics have been noted to cause fever, with clozapine being the most notable. In reference to clozapine, fever is most commonly seen within the first 3 weeks of therapy, and has a very wide reported incidence (0.5–55%) [26, 30, 31]. While clozapine-induced fevers are generally noted to be transient (lasting between 2 and 4 days on average) and benign, they may necessitate discontinuation, or at the very least further investigation to rule out alternative pathophysiology [26, 27, 32]. In the instance where clozapine is restarted after discontinuation, case reports determine it is well-tolerated with no further episodes of fever; however, Chung et al. found 1 out of 7 patients (14.3%) had fever on rechallenge [28, 33]. It is unclear if fever from clozapine is related to dose or the rate of titration, though it has been noted to occur with titration rates of > 50 mg/week, and when given concomitantly with valproic acid [28, 31]. Studies have demonstrated that the pro-inflammatory cytokine interleukin-6 may have a specific role in mediating clozapine-induced fevers [31].

Less well known, the pathophysiology behind antipsychotic-induced hypothermia appears complex, and multiple mechanisms have been proposed. Clozapine possesses a unique pharmacologic profile that encompasses activity on numerous receptors, including antagonistic effects on D_2_, D_3_, alpha-1, 5-HT_2A/C_, and M_3_, and partial agonistic effects on 5-HT_1A_. N-desmethylclozapine (NDMC), unlike its parent compound, possesses D_2_ and D_3_ partial agonistic effects, as well as M_3_ agonism [34, 35]. Table 4 summarizes literature on mice (or rat) studies conducted with various unique compounds possessing similar or opposite effects as clozapine on these receptors. Intrinsic efficacy of clozapine on D_1_ receptors appears to show it may be an agonist, rather than antagonist, at this receptor [54]. If this were true, it would suggest D_1_ agonism as a cause of hypothermia. Mitigation of clozapine-induced hypothermia by the D_1_ antagonist SCH-23390 supports this hypothesis [37]. Antagonistic effects on D_2_ do not support clozapine-induced hypothermia, but may be supported by the agonistic effects of NDMC on this receptor, as the D_2_ agonist bromocriptine has been shown to produce hypothermia in mice [34, 38, 39]. Similarly, hypothermic effects by norclozapine have been mimicked by the D_2/3_ agonist (+)-7-OH-DPAT, and blocked by the D_3_-selectve antagonist (+/−)-S 11566 in rats [40]. However, studies in D_3_ knockout mice have indicated that this effect may not be secondary to D_3_ agonism [41]. 5-HT_2A/C_ antagonism corroborates potential hypothermic effects from clozapine, as agonists of this receptor (MK-212 and DOI) have been shown to reverse the hypothermic effects of chlorpromazine and haloperidol (antagonists of this receptor) [47]. Conversely, because clozapine is thought to possess 5-HT_7_ inverse agonist properties, the hypothermic effects seen with the selective 5HT_7_ receptor agonist LP-44 may disprove any implication of this receptor [50]. Minimal data exists on the effects of M_3_-selective agonists on temperature dysregulation, but if one were to consider the hyperthermic effects of M_3_ antagonists such as hyoscine, then it may stand to reason that the opposite effects potentially induce hypothermia [52]. Inhibition of compensatory responses to hypothermic reactions, such as shivering and vasoconstriction, through the peripheral alpha-1 antagonistic properties of clozapine should also be considered as an exacerbating, or possibly primary, factor [43]. Lastly, it has been shown that all antipsychotics increase neurotensin levels within the rodent brain [55, 56]. Central agonistic effects of neurotensin induce hypothermia, while peripheral effects may induce hypotension [55, 56]. Given that clozapine and NDMC bind to a multitude of receptors, it is possible that no single mechanism is responsible, but rather may be caused by an interplay of activity tilting the balance in favor of hypothermia. However, because other antipsychotics not possessing the same receptor profile as clozapine or NDMC have been noted to cause hypothermia, a reasonable assumption would be the commonality of 5HT_2A_ antagonism or neurotensin-1 augmentation amongst these agents as the primary pathophysiology.

**Table 4:** Receptor/Ligand Data Supporting or Refuting Propensity of Clozapine & NDMC to Induce Hypothermia

Receptor	Author, year	Conclusions
**D**_**1**_	Ogren SO, 1988 [36] Salmi P, 1994 [37]	The D_1/2_ receptor agonist apomorphine and the D_2_ agonist pergolide induced hypothermia in rats, which was prevented by the use of sulpiride, a D_2_ antagonist.
		Hypothermia produced by clozapine was fully antagonized by the selective D_1_ receptor antagonist SCH-23390.
**D**_**2**_	Zarrindast MR, 1989 [38] Boulay D, 1999 [39]	Bromocriptine, a D_2_ agonist, caused dose-dependent decreases in the core body temperature of mice. This effect was mitigated by pretreatment with sulpiride
		The preferential D_2/3_ receptor agonists 7-OH-DPAT and PD 128907 induced hypothermia in D_2_ (+/+) mice, but not in D_2_ knockout mice.
**D**_**3**_	Millan MJ, 1995 [40] Perachon S, 2000 [41] Varty GB, 1998 [42]	Similar to (+)-7-OH-DPAT, clozapine dose-dependently elicited hypothermia in rats. The D_3_-selective antagonist (+/−)-S 11566 blocked clozapine-induced hypothermia.
		(+)-7-OH-DPAT was effective in inducing hypothermia in both D_3_ (+/+) and D_3_ knockout mice, suggesting the D_3_ receptor is not responsible for hypothermia.
		Raclopride (D_2/3_ antagonist) blocked (+)-7-OH-DPAT induced hypothermia.
**Alpha-1**	Boschi G, 1987 [43]	Phenothiazines, butyrophenones, and benzamides (alpha-1 antagonists) injected intraperitoneally demonstrated induced-hypothermia, whereas intracerebovascular administration did not. The administration of phenylephrine (alpha-1 agonist) attenuated hypothermia.
**5HT**_**1A**_	Gudelsky GA, 1986 [44] Abdel-Fattah AF, 1995 [45] Neves G, 2008 [46]	The 5HT_1A_ agonist 8-OH-DPAT induced dose-related decreased in temperature in rats.
		Pindolol, a 5HT_1A_ antagonist, suppressed tryptophan (serotonin precursor) induced hypothermia in pargyline-treated mice.
		The hypothermia produced by the N-phenylpiperazine derivatives LASSBio-579 and LASSBio-581 was diminished by the 5-HT_1A_ antagonist WAY 100635.
**5HT**_**2A/C**_	Yamada J, 1995 [47] Murphy TJ, 2019 [48] Gudelsky GA, 1986 [44]	The centrally acting 5HT_2A/C_ agonist I-2,5-dimethoxy-4-iodophenyl)-2-aminopropane (DOI) strongly inhibited haloperidol and chlorpromazine-induced hypothermia.
		The selective 5HT_2C_ agonist WAY-163909 inhibited ketamine-induced hypothermia, whereas DOI did not.
		MK-212 (5HT_2_ agonist) induced hyperthermia, while mianserin (5HT_2_ antagonist) blocked hyperthermia caused by MK-212 in rats.
**5HT**_**7**_	Hedlund PB, 2010 [49] Naumenko VS, 2011 [50]	LP-211 (5HT_7_ selective agonist) induced hypothermia in 5HT_7_ (+/+) mice, but not 5HT_7_ receptor knockout mice.
		The selective 5HT_7_ receptor antagonist SB 269970 inhibited centrally administered LP-44 (5HT_7_ agonist) induced hypothermia. Intraperitoneal administration of LP-44 did not induce hypothermia.
**M**_**3**_	Black CE, 2001 [51] Golding JF, 2018 [52]	M_3_ antagonism via hyoscine hydrobromide may induce hyperthermia through decreased skin conductance and vasoconstriction, reducing heat loss and sweating.
**Neurotensin-1 receptor (NTS-1)**	Feifel D, 2010 [53]	PD149163, a selective, brain-penetrating, NT1 receptor agonist produced hypothermia in rats.

## Conclusions

Clinicians should be cognizant of the risk of hypothermia from clozapine, which can be severe or even fatal. Frequent temperature monitoring should be performed during initiation and titration of an antipsychotic, especially in those with comorbid CKD or dementia, or concomitantly taking benzodiazepines, valproic acid, or additional antipsychotics. This effect requires additional investigation, as multiple pharmacologic mechanisms are proposed.

## Data Availability

Data sharing is not applicable to this article, as this is a single-patient case report. No datasets besides those reported in the article were generated during the current study.

## Abbreviations

CKD: Chronic kidney disease
FDA: Food and Drug Administration
WHO: World Health Organization
CYP450: Cytochrome P450
NMS: Neuroleptic malignant syndrome
NDMC: N-desmethylclozapine
TSH: Thyroid stimulating hormone
SUD: Sudden unexpected death
